# 1690. Assessing the safety of TP-102 bacteriophage treatment in the management of diabetic foot infections

**DOI:** 10.1093/ofid/ofac492.1320

**Published:** 2022-12-15

**Authors:** Ran Nir-Paz, Hadil Onallah, Yechiel N Gellman, Amir Haze, Ron Braunstein, Ronen Hazan, Clara Leandro, Raquel Barbosa, Helena Dordio, Alexandra Wagner, Sofia V Corte Real, Miguel Garcia

**Affiliations:** Hadassah-Hebrew University Medical Center, Jerusalem, Yerushalayim, Israel; Hadassah-Hebrew University Medical Center, Jerusalem, Yerushalayim, Israel; 1Hadassah-Hebrew University Medical Center, Jerusalem, Yerushalayim, Israel; 1Hadassah-Hebrew University Medical Center, Jerusalem, Yerushalayim, Israel; Hebrew University of Jerusalem, Jerusalem, Yerushalayim, Israel; The Hebrew University, Jerusalem, Yerushalayim, Israel; Technophage, Investigação e Desenvolvimento em Biotecnologia S.A., Lisbon, Lisboa, Portugal; Technophage, Investigação e Desenvolvimento em Biotecnologia S.A., Lisbon, Lisboa, Portugal; Technophage, Investigação e Desenvolvimento em Biotecnologia S.A., Lisbon, Lisboa, Portugal; Technophage, Investigação e Desenvolvimento em Biotecnologia S.A., Lisbon, Lisboa, Portugal; Technophage, Investigação e Desenvolvimento em Biotecnologia S.A., Lisbon, Lisboa, Portugal; Technophage, Investigação e Desenvolvimento em Biotecnologia S.A., Lisbon, Lisboa, Portugal

## Abstract

**Background:**

Diabetic Foot Ulcers (DFU) are a major complication in patients with diabetes and affect globally 170 million patients. Approximately 28% of patients with infected DFU require amputation. Diabetic foot infections (DFI) caused by persistent or multidrug resistant organisms are a threat to the outcome of therapy. Infections are mostly polymicrobial, and major pathogens include *Staphylococcus aureus* and *Pseudomonas aeruginosa*. A promising new approach using phages may potentially be a novel modality to increase treatment success. TP-102 is an innovative bacteriophage cocktail for treatment of DFI, a diabetes related unmet need.

**Methods:**

TP-102 comprised of 5 different phages, targeting *S. aureus*, *P. aeruginosa* and *Acinetobacter baumannii*. This study is a Phase I/II clinical trial performed in Israel looking at safety and tolerability. TP-102 bacteriophage cocktail was applied topically at a titer of 10^9^ PFU/mL/cm^3^ of target ulcer. Two cohorts receive multiple doses of TP-102. In non-infected DFU (cohort 1) for 1 week and for 28 days in DFU with infection grade 2 or 3 (PEDIS classification, cohort 2). Treatment groups were TP-102 with standard of care versus placebo and standard of care. Outcomes include microbiologic data on the 3 target bacteria, ulcer healing characteristics, incidence and severity of TP-102-emergent solicited local and systemic adverse events, and their relationship to the treatment, from the first application of TP-102 until the end of study.

**Results:**

For the evaluation of safety in patients with non-infected ulcers, eight patients were enrolled to the first cohort. Six were treated with TP-102 and 2 with placebo. TP-102-treated patients had no severe adverse events associated with the treatment. In the second cohort a total of 18 patients are currently being treated with TP-102 or placebo for 28 days, three times a week. Recruitment is ongoing and results of the trial will be presented.

**Conclusion:**

TP-102 appeared to be safe when applied to patients with uninfected diabetic foot ulcers. The treatment is a potential new non-traditional antimicrobial for topical application. The efficacy of TP-102 in the treatment of infected diabetic ulcers is being assessed in an ongoing randomized clinical trial.

**Disclosures:**

**Ran Nir-Paz, MD**, BiomX: Advisor/Consultant|Technophage: Advisor/Consultant **Clara Leandro, PhD**, Technophage: Employee **Raquel Barbosa, PhD**, Technophage: Employee **Helena Dordio, PhD**, Technophage S.A.: Employee **Miguel Garcia, n/a**, IBV Innovation Bioventures Lda: Board Member|IBV Innovation Bioventures Lda: Ownership Interest|LX Bio Pharmaceuticals SA: Board Member|LX Bio Pharmaceuticals SA: Ownership Interest|TechnoPhage, SA: Board Member|TechnoPhage, SA: Author of several patent applications|TechnoPhage, SA: Ownership Interest.

